# Novel human monoclonal antibodies targeting the F subunit of leukocidins reduce disease progression and mortality caused by *Staphylococcus aureus*

**DOI:** 10.1186/s12866-018-1312-7

**Published:** 2018-11-12

**Authors:** Chendi Jing, Chenghua Liu, Fangjie Liu, Yaping Gao, Yu Liu, Zhangchun Guan, Bo Xuan, Yanyan Yu, Guang Yang

**Affiliations:** 1 Department of Infectious Diseases, Peking University First Hospital, Beijing, 100034 China; 2 Beijing Institute of Basic Medical Sciences, Beijing, 100850 China; 3 State Key Laboratory of Toxicology and Medical Countermeasures, Beijing, China

**Keywords:** *Staphylococcus aureus*, Leukocidins, Human monoclonal antibodies

## Abstract

**Background:**

*Staphylococcus aureus* is a leading cause of Gram-positive bacterial infections worldwide; however, the treatment of *S. aureus* infection has become increasingly difficult due to the prevalence of methicillin-resistant *S. aureus* strains, highlighting the urgent need for the development of novel strategies. The complexity of *S. aureus* pathogenesis relies on virulence factors. Recent studies have demonstrated that leukocidins expressed by the majority of clinical isolates play important roles in the pathogenesis of *S. aureus*.

**Results:**

In this study, we developed three human monoclonal antibodies against all F-components of leukocidins HlgABC, LukSF, and LukED with high affinity. These antibodies were found to be capable of blocking leukocidin-mediated cell lysis in vitro. Furthermore, the antibodies dramatically reduced disease progression and mortality after *S. aureus* infection in vivo.

**Conclusions:**

Our findings revealed that neutralizing bicomponent leukocidins may be a promising strategy to combat infections caused by *S. aureus.*

**Electronic supplementary material:**

The online version of this article (10.1186/s12866-018-1312-7) contains supplementary material, which is available to authorized users.

## Background

*Staphylococcus aureus* is a Gram-positive bacterium that is responsible for significant morbidity and mortality worldwide [1]. *S. aureus* causes a wide range of infections, including mild skin infections, bacteremia, sepsis, endocarditis, and pneumonia [2]. Antibiotic treatment of *S. aureus* infections has become increasingly difficult owing to the emergence of methicillin-resistant *S. aureus* strains, emphasizing the need for alternative, nonantibiotic options to combat this pathogen, such as human monoclonal antibodies (mAbs) directed against virulence factors [3, 4].

*S. aureus* express five different membrane-damaging toxins: four hemolysins (alpha-, beta-, gamma-, and delta-hemolysin) and leucocidins. γ-hemolysin can efficiently damage host defense cells and red blood cells [5, 6], thereby playing an important role in evasion of the innate immune response [7–10]. Moreover, γ-hemolysin contributes partially to virulence during septic arthritis and systemic infection in mice [11, 12] and endophthalmitis in rabbits [13, 14]. γ-hemolysin forms two functional bicomponent (S and F component) toxins (HlgAB and HlgCB), which share the F component HlgB [5].

To date, several other bicomponent (S and F component) toxins LukED, LukSF-PV/PVL, and LukAB/HG, have been shown to be involved in the pathogenesis of *S. aureus* [7–9].

γ-hemolysin and leucocidins belong to pore-forming toxins [15]. The S component can bind to cellular receptors and induce conformational change to allow dimerization with F components [16]. These dimers then oligomerize to form the pre-pore prior to insertion of the β-barrel transmembrane channel [17]. Recent studies demonstrated that γ-hemolysin is produced by more than 99.5% of human *S. aureus* isolates, other leukocidins is not as widely distributed but implicated in the manifestation of more severe disease [18, 19].

In the present study, we aimed to identify neutralizing monoclonal antibodies (mAbs) against HlgB that could block γ-hemolysin cytotoxicity. From our analysis, we discovered three human mAbs targeting HlgB that crossrecognized the F components of leukocidins and blocked *S. aureus* infection.

## Results

### Rabbit red blood cells (RBCs) and human leukocytes were susceptible to γ-hemolysin

**Fig. 1:**
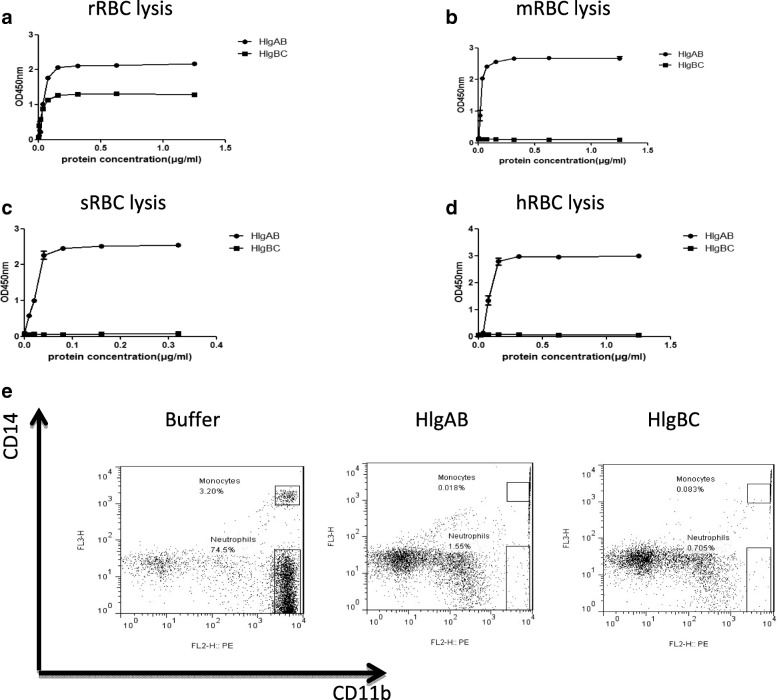
Rabbit RBCs and human leukocytes were susceptible to γ-hemolysin. **(a)** HlgAB and HlgBC induced the hemolysis of rabbit red blood cells (rRBCs) in a concentration-dependent manner. **(b–d)** HlgAB induced the hemolysis of mouse red blood cells (mRBCs) **(b)**, sheep red blood cells (sRBCs) **(c)**, and human red blood cells (hRBCs) **(d)** in a concentration-dependent manner. Serial dilutions of HlgAB and HlgBC proteins were incubated with 2% (*v*/v) solution of erythrocytes at 37 °C for 1 h. The samples were centrifuged, and the absorbance of the supernatants was measured at 405 nm. **(e)** HlgAB and HlgCB efficiently induced cell lysis of human neutrophils and monocytes. Leukocytes were isolated from a healthy donor, incubated with PBS or γ-hemolysin at room temperature for 1 h, and gated for CD14 and CD11b positivity. The percentages of human neutrophils and monocytes were determined by FCM

The F component (HlgB) and two S components (HlgA, HlgC) of γ-hemolysin were expressed and analyzed by SDS-PAGE and Coomassie blue staining (Additional file 1: Figure S1). The sensitivity of RBCs from different species (rabbits, mice, sheep, and humans) to γ-hemolysin was determined by incubation with recombinant γ-hemolysin (HlgAB or HlgBC) at 0.01–5 μg/mL. HlgAB was found to efficiently lyse RBCs from all four species. However, only rRBCs were sensitive to lysis mediated by HlgBC (Fig. 1a–d). Human leukocytes are known to be sensitive to killing by γ-hemolysins [20]. Therefore, we further detected the activities of HlgAB and HlgBC in human leukocytes. We found that human neutrophils and monocytes were more susceptible to both HlgAB and HlgBC killing than lymphocytes (Fig. 1e).

### Selection of anti-HlgB human mAbs

We screened a naïve human Fab phage library to select antibodies that specifically bound HlgB. After three rounds of panning, 168 phage clones were further evaluated by ELISA, and 16 clones (OD_450nm_ ≥ 0.8) were then subjected to nucleotide sequencing (Additional file 2: Figure S2). Three antibodies that specifically bound to HlgB were isolated from the library and designated YG8–1, YG8–2, and YG8–3.

**Fig. 2:**
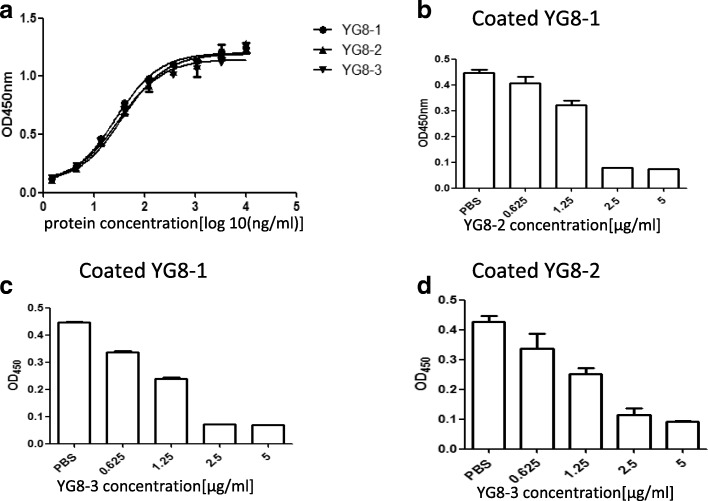
YG8–1, YG8–2, and YG8–3 bound to the same region of HlgB. **(a)** The binding affinities of YG8–1, YG8–2, and YG8–3 to HlgB were determined by ELISA. HlgB was coated onto 96-well plates in the presence of serial dilutions of YG8–1, YG8–2, and YG8–3. Bound IgG was detected using horseradish peroxidase (HRP)-conjugated goat anti-human IgG. **(b–d)** YG8–1, YG8–2, and YG8–3 competed with each other in a concentration-dependent manner. YG8–1 was coated onto 96-well plates, and YG8–2 **(b)** or YG8–3 **(c)** was added at various concentrations after pre-incubated with GST-HlgB. YG8–2 was coated onto 96-well plates, and YG8–3 **(d)** was added at various concentrations after pre-incubation with GST-HlgB. Bound GST-HlgB was detected using an anti-GST antibody. Data are representative of three independent experiments and shown as the mean ± SD

To test the functions of YG8–1, YG8–2, and YG8–3, these mAbs were expressed and purified as full-length human IgG1 antibodies (Additional file 3: Figure S3). The binding affinities of YG8–1, YG8–2, and YG8–3 to HlgB were subsequently measured by ELISA and surface plasmon resonance (BIAcore technology). Interestingly, YG8–1, YG8–2, and YG8–3 had comparable binding affinity in the picomolar range (Fig. 2a, Additional file 4: Figure S4, and Table 1). We further determined whether YG8–1, YG8–2, and YG8–3 had the same binding site in HlgB. Competitive ELISA performed with GST-HlgB revealed that all three mAbs competed with each other in a concentration-dependent manner, suggesting that they bound to the same region of HlgB (Fig. 2b–d).

**Table 1:** Binding affinities of YG8–1, YG8–2 and YG8–3 for F-components as determined by ELISA and Surface Plasmon Resonance analyses

Antigen	Antibody	EC50(ng/ml)	K_D_
HlgB	YG8–1	27.07 ± 1.12	4.181E-12
	YG8–2	40.22 ± 5.64	4.916E-12
	YG8–3	31.16 ± 1.12	1.554E-11
LukF	YG8–1	15.57 ± 1.41	1.240E-13
	YG8–2	20.25 ± 1.99	2.312E-13
	YG8–3	15.92 ± 1.29	1.279E-13
LukD	YG8–1	25.34 ± 4.91	1.381E-10
	YG8–2	46.07 ± 15.50	1.344E-10
	YG8–3	23.33 ± 2.24	2.211E-10

Data are representative of three independent experiments and shown as the mean ± SD

### YG8–1, YG8–2, and YG8–3 bound to all F components

**Fig. 3:**
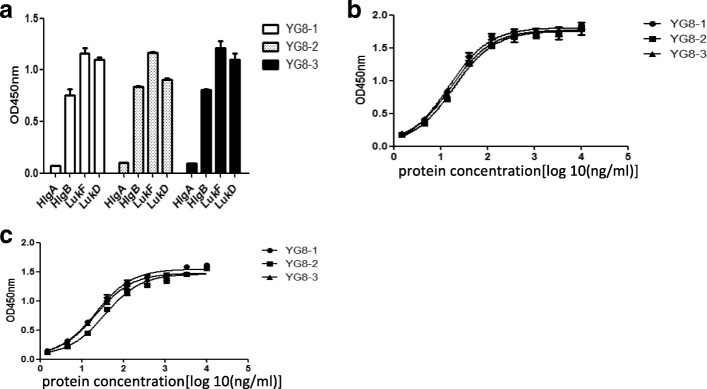
YG8–1, YG8–2, and YG8–3 bound to all F components. **(a)** YG8–1, YG8–2, and YG8–3 bound to HlgB, LukF, and LukD, but not HlgA. HlgA, HlgB, LukF, and LukD were coated (1 μg/well) onto 96-well plates. Bound IgG was detected using horseradish peroxidase (HRP)-conjugated goat anti-human IgG. Data are presented as the mean ± SD of three independent experiments. **(b, c)** The binding affinities of YG8–1, YG8–2, and YG8–3 to LukF **(b)** or LukD **(c)** were determined by ELISA. LukF or LukD was coated onto 96-well plates in the presence of serial dilutions of YG8–1, YG8–2, and YG8–3. Bound IgG was detected using horseradish peroxidase (HRP)-conjugated goat anti-human IgG

Given the high amino acid conservation among HlgB, LukF, and LukD (Additional file 5: Figure S5), we examined whether YG8–1, YG8–2, and YG8–3 bound to not only HlgB but also other F components. The recombinant toxin molecules (LukS, LukF, LukE, and LukD) were expressed and generated (Additional file 1: Figure S1). To ensure high quality and functionality of protein baits, toxins were tested for cytolytic activity in vitro assays. The leukocidins were tested with freshly isolated human leukocytes. We observed that LukSF and LukED lysed human neutrophils and monocytes with high potency (data not shown). To determine whether these HlgB-reactive mAbs interacted with other F components, the binding of these mAbs to LukF and LukD was measured by ELISA. The results showed that YG8–1, YG8–2, and YG8–3 could bind to LukF and LukD but did not interact with HlgA (Fig. 3a). Most importantly, we found that YG8–1, YG8–2, and YG8–3 had very high affinity for LukF and LukD (Fig. 3b and c, Additional file 4: Figure S4, and Table 1).

### Detection of the neutralization function of mAbs in vitro

**Fig. 4:**
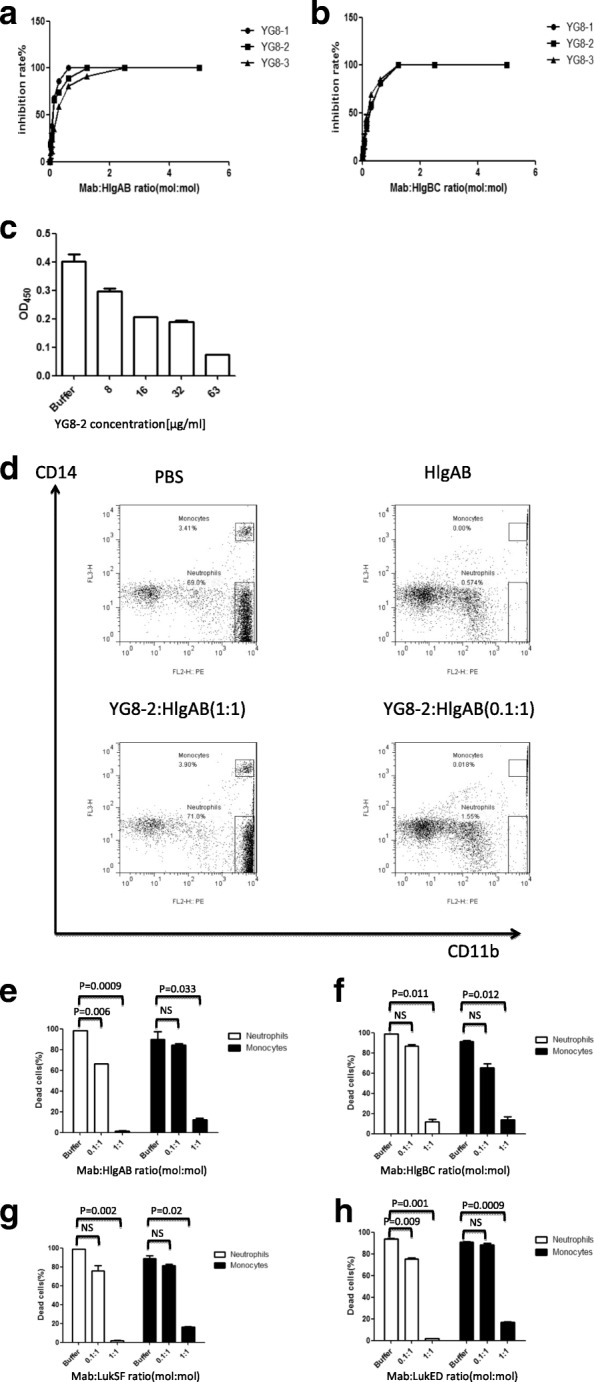
Neutralization ability of YG8–1, YG8–2, and YG8–3 in vitro. **(a, b)** YG8–1, YG8–2, and YG8–3 inhibited the hemolysis of rabbit red blood cells (rRBCs) in a concentration-dependent manner. YG8–1, YG8–2, and YG8–3 were serially diluted in PBS and mixed with recombinant HlgAB **(a)** or HlgBC **(b)**. Pre-incubation was carried out at room temperature for 30 min, and the mAbs were then incubated with erythrocytes at 37 °C for 1 h. The samples were centrifuged, and the absorbance of the supernatants was measured at 405 nm. Percent inhibition of toxin activity was calculated using the following formula: percent inhibition = ([normal activity – inhibited activity] / [normal activity]) × 100. **(c)** YG8–2 inhibited the interaction between HlgA and HlgB. His-HlgA (1 μg/well) was coated onto 96-well plates, and YG8–2 was added at various concentrations after pre-incubation with GST-HlgB. Bound GST-HlgB was detected using an anti-GST antibody. Data are representative of three independent experiments and shown as the mean ± SD. **(d–h)** YG8–2 inhibited the lysis of neutrophils and monocytes in a concentration-dependent manner. YG8–2 was pre-incubated with HlgAB **(d, e)**, HlgBC **(f)**, LukSF **(g)**, and LukED **(h)** at various concentrations at room temperature for 30 min, and the mixture was then incubated with leukocytes at room temperature for 1 h, followed by gating for CD14 and CD11b positivity. The percentages of human neutrophils and monocytes were determined by FCM. Representative results **(d)** and statistical analysis **(e–h)** of three independent experiments are presented. Data are shown as the mean ± SD. Significant differences between groups were evaluated using two-tailed Student’s t tests

Since rabbit RBCs were significantly more sensitive to HlgAB and HlgBC, we evaluated the neutralizing abilities of these mAbs toward rabbit RBCs treated with HlgAB and HlgBC. Antibody potency was expressed as half-maximal inhibition of cell lysis (IC_50_). In these assays, we found that YG8–1, YG8–2, and YG8–3 efficiently neutralized HlgAB and HlgBC in rabbit RBCs (Fig. 4a and b). The neutralization potencies of these mAbs were highly comparable (Table 2). Because the three antibodies recognized the same region of HlgB and the production of YG8–2 was higher than that of YG8–1 and YG8–3, we used YG8–2 in subsequent neutralization assays. As a bicomponent toxin, the activity of γ-hemolysin was found to be dependent on the formation of the heterodimer [17, 21]. The results of competitive ELISA demonstrated that YG8–2 blocked the interaction between HlgA and HlgB (Fig. 4c).

**Table 2:** The neutralization potencies of YG8–1, YG8–2, and YG8–3 toward rabbit RBCs treated with HlgAB and HlgBC. IC50 expressed as mAb:toxin ratio

toxin	HlgAB	HlgBC
mAbs		
YG8–1	0.10	0.28
YG8–2	0.12	0.27
YG8–3	0.26	0.21

In further experiments, we investigated whether YG8–2 could prevent lysis of human leukocytes by leukocidins (HlgABC, LukSF, and LukED). The neutralization potency toward leukocidins was demonstrated using in vitro assays when human leukocytes were treated with a mixture of recombinant leukocidin at a concentration that could induce more than 90% cell lysis. We found that YG8–2 also neutralized the cytotoxic activities of leukocidins in a concentration dependent manner. (Fig. 4d–h).

### Detection of the neutralization ability of mAbs in vivo

**Fig. 5:**
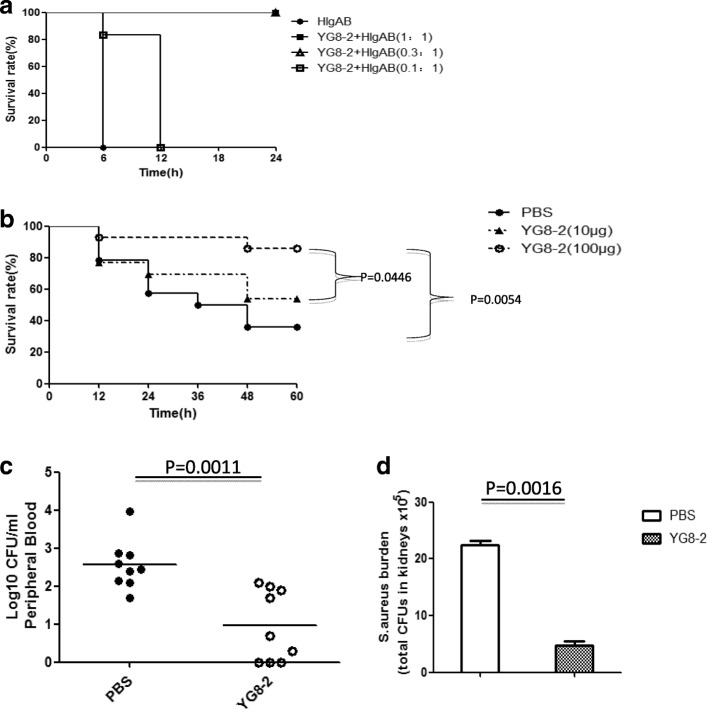
Neutralization ability of YG8–2 in vivo**. (a)** The neutralization potency of YG8–2 was determined in retroorbital challenge of mice with HlgAB. YG8–2 was serially diluted and mixed with HlgAB (10 μg/mL) at room temperature for 30 min, and the mixture (100 μL) was then injected into mice. The survival rate was measured at different time points postchallenge. Data are presented as the percentage of mice surviving. Survival curves were plotted using the Kaplan-Meier method and compared using the log-rank test (*n* = 6). **(b–d)** Determination of the effects of YG8–2 in a murine peritonitis model. Different concentrations of YG8–2 (10 or 100 μg/mouse) were injected into the peritoneal cavity of BALB/c mice 4 h prior to intraperitoneal challenge with *S. aureus* USA300 (0.75 × 10^8^ CFU/mouse). The survival rate was measured at different time points postchallenge **(b)**. At 12 h postinfection, peripheral blood was plated to evaluate the bacterial burden **(c)**. The surviving mice were sacrificed, and bacterial colonies in the kidneys were counted at 60 h postinfection **(d)**. Data are representative of three independent experiments and shown as the mean ± SD. Significant differences between groups were evaluated using two-tailed Student’s t tests. Survival curves were determined using the Kaplan-Meier method and compared using the log-rank test (*n* = 14)

The protective efficacy of YG8–2 was evaluated in a γ-hemolysin attacking model. In this model, animals were randomized for retroorbital challenge with lethal doses of HlgAB or HlgAB pre-incubated with different concentrations of YG8–2. Survival rates were monitored for 24 h (Fig. 5a). We found that pre-incubation with YG8–2 dramatically reduced mortality rates.

Next, we investigated the contribution of this neutralizing ability to *S. aureus* pathogenesis in vivo in a murine peritonitis model. In this model, YG8–2 was injected (10 or 100 μg/mouse) into the peritoneal cavity of BALB/c mice 4 h before intravenous challenge with a lethal dose of *S. aureus* USA300 (0.75 × 10^8^ CFU/mouse). The survival rates were 36% (5/14) for animals pretreated with control buffer and 50% (7/14) for animals pretreated with a low concentration of YG8–2; in contrast, in animals pretreated with a high concentration of YG8–2, the survival rate was 86% (12/14; Fig. 5b). This result suggested that neutralization of bicomponent leukocidins enhanced survival rates in a murine peritonitis model.

Staphylococcal peritonitis is used as a model to study the spread of bacteria in the bloodstream [22]. We found that the number of viable bacteria in the peripheral blood of mice was dependent on YG8–2 administration. Twelve hours after intraperitoneal infection, control mice infected with *S. aureus* USA300 showed an increase in the number of viable bacteria by about 30 fold in the peripheral blood compared with that in mice pretreated with YG8–2 (100 μg/mouse; Fig. 5c). In addition, the protective effects of YG8–2 were observed based on the number of bacterial colonies in the kidneys (Fig. 5d).

## Discussion

The evolution of antibiotic-resistant *S. aureus* strains has been a great challenge to global public health and highlights the urgent need for novel preventive and theraputic strategies. Multidrug-resistant strains have enhanced virulence potential owing to their ability to secrete leukocidins, which comprise a class of bicomponent pore-forming toxins capable of damaging host immune cells [7, 8, 10]. Targeting of leukocidins is regarded as a promising strategy that would increase the host immune response to defense against *S. aureus* infection [23].

In this study, we generated a human monoclonal antibody that bound F-components of leukocidins HlgABC, LukSF, and LukED, thereby blocking leukocidin-mediated evasion of phagocytosis and dramatically reducing *S. aureus* infection. Using a naïve human Fab phage library, we obtained three mAbs against HlgB. We showed that these mAbs bound to the same epitope of HlgB and had comparable neutralizing potency. Importantly, recent studies have demonstrated that the binding affinities of mAbs for toxins are highly predictive of neutralization potency and efficacy [24, 25]. YG8–1, YG8–2, and YG8–3 have high dissociation constants (K_D_) between 2.21 × 10^10^ mol^− 1^ and 1.24 × 10^13^ mol^− 1^ as measured by surface plasmon resonance (SPR), and it is likely that these mAbs would provide good protection against toxins. Furthermore, we found that YG8–1, YG8–2, and YG8–3 could bind to all F components of leukocidins, except LukAB, which shares less than 40% identity with HlgB.

In our in vitro studies, we found that YG8–2 could prevent lysis of human leukocytes by leukocidins (HlgAB, HlgBC, LukSF, and LukED). Furthermore, we demonstrated that YG8–2 could inhibit the interaction between HlgA and HlgB and consequently block their cytotoxic ability in vitro and in vivo. Based on studies demonstrating that leukocidins target cells in a species-specific manner [26–28], the implementation of murine models to study YG8–2 neutralization potency is limited. Using a murine peritonitis model, we observed that mice pretreated with YG8–2 (100 μg/mouse) showed a 50% increase in survival in the bacteremia model compared with that in the control group. In addition, we identified YG8–2 as a protective factor alleviating bacteremia and the number of bacterial colonies in the kidneys, these observations strongly suggested that YG8–2 protected mice against challenge with antibiotic-resistant strains of *S. aureus*. Recently, many studies have addressed the contributions of leukocidins to the pathogenesis of *S. aureus* in murine models [20, 26, 28, 29]; however, the results have been unclear. Interestingly, in our study, administration of YG8–2 greatly reduced virulence in a murine peritonitis model. Previous studies demonstrated that HlgAB and LukED could target murine neutrophils and monocytes [20, 28, 29]; thus, YG8–2 may exert beneficial effects by neutralizing multiple leukocidins. We suspect that in addition to inhibiting the formation of cytolytic pores, YG8–2 may also block the interaction between S components and their unique receptors, thereby neutralizing the cytotoxic capacity. Further studies are needed to address this hypothesis. In this study, the preventive effect of YG8–2 was determined by administration of antibody before *S.aureus* challenge. However, the therapeutic effect of YG8–2 needs to be investigated in the future.

Several mAbs have been made to neutralizing α-hemolysin and leucocidins [30–34]. Rouha et al. showed that a single Hla-LukF-LukD-HlgB crossreactive antibody could deactivate five potent cytolysins and provide improved protection against rabbit lethal pneumonia compared with the Hla-specific mAb [24, 35]. Moreover, a combination of two monoclonal antibodies (named as ASN100) that target α-hemolysin and leucocidins is being tested in a Phase 2 clinical trial for the prevention of *S. aureus* pneumonia in mechanically ventilated patients (NCT02940626) [34]. Thus, we suggest that therapeutic administration of YG8–2 in combination with other neutralizing mAbs or antibiotics may provide a possible strategy to improve treatment outcomes.

## Conclusions

We have shown that leukocidins play important roles in *S. aureus* pathogenesis. Targeting of F components using the neutralizing antibodies generated herein may be an effective therapeutic strategy for the treatment of infectious diseases caused by *S. aureus.*

## Material and methods

### Bacterial strains and growth conditions

The *E.coli* strains BL21 and DH5α were purchased from Novagen. The *S. aureus* strain USA 300 was kindly provided by professor Lefu Lan (Department of Molecular Pharmacology, Shanghai Institute of Materia Medica, Chinese Academy of Sciences). *E.coli* cells were grown in Luria-Bertani (LB) medium and *S. aureus* cells were grown in brain heart infusion (BHI) medium at 37 °C for 12 h with shaking at 220 rpm.

### Protein purification and generation

Recombinant proteins fused 6 × His at the N terminus, including HlgA, HlgB, HlgC, LukS, LukF, LukE, LukD were expressed in *E.coli* (BL21) and purified using Ni-NTA agarose (GE Healthcare, 17–5318-01) and dialyzed in PBS for 24 h at 4 °C. GST fusion proteins (GST-HlgB) were expressed in *E.coli* (DH5α) and purified using glutathione agarose (Transgene, DP201–01) according to manufacturer’s instructions.

### RBC hemolysis assay

The RBC hemolysis assay was performed as described previously with some modifications [36, 37]. In brief, the serial dilution of HlgAB and HlgBC proteins were incubated with 2% (*v*/v) solution of erythrocytes at 37 °C for 1 h. After incubation, the supernatant of cells was collected by centrifugation (860 g for 10 min) and then measured at 405 nm.

### Human leukocytes lysis assay

Human leukocytes were collected as described previously [36], then incubated with different concentration of Leukocins (HlgAB, HlgBC, LukSF and LukED) at room temperature (RT) for 1 h. After incubation, cells were washed twice with PBS, then stained for 30 min at RT with PE-conjugated CD11b (Biolegend, 101,207) and PerCP-conjugated CD14 (Biolegend, 325,632). The stained cells were analyzed by flow cytometry (BD FACSCalibur, San Jose, USA), and the percentage of survival cells from each sample was calculated.

### Selection of monoclonal antibodies

HlgB specific antibodies were isolated from a phage display antibody library using the standard procedure [38]. In brief, HlgB was coated on Nunc-immunotube (Thermo Scientific Nunc®, 444,202) at 4 °C overnight. Then phages (10^12^ CFU/mL) were incubated with HlgB for 1 h, unbound phages were removed. The bound phages were eluted using 0.1 M Gly-HCl (pH 2.2). *E. coli* TG1 cells were infected with the eluted phages. The amplified phages were then subjected to the next round of panning. After three rounds of panning, single colonies were randomly selected and further screened by ELISA. The sequences of the specific phage clones binding to HlgB were analyzed.

### Binding affinity of mAbs

Binding affinity of antibodies (YG8–1, YG8–2 and YG8–3) to F components of leukocidins was determined by ELISA. Briefly, HlgB, LukF, and LukD were coated individually overnight at 4 °C, then incubated with serially diluted antibodies (YG8–1, YG8–2 and YG8–3) at 37 °C for 1 h. The bound antibody was detected using horseradish peroxidase/Goat anti-Human antibody (1:40000; Jackson, 109–035-003).

Binding affinity was also determined by surface plasmon resonance using a Biacore X100 instrument. Anti-human IgG antibody was immobilized on the carboxymethylated dextran surface (BIAcore’s CM5 chip) using the human antibody capture kit (GE Healthcare, BR100839), then antibodies (YG8–1, YG8–2 and YG8–3) were captured. Proteins were serially diluted to different concentration (0.625–20 μg/mL) in the running buffer and incubated with antibody bound on the tips. Association and dissociation phase were measured for 180 s and 1300s respectively. The data were analyzed using the Biacore X100 Evaluation software.

### Competitive ELISA

The 96-well plates were coated with YG8–1 overnight. After blocking with 5% non-fat milk at 37 °C for 1 h, serially dilutions of YG8–2 and YG8–3 were added to the wells respectively, which mixed with 10 μg/mL GST-HlgB, after incubated at 37 °C for 1 h and washed 5 times with PBST, the monoclonal mouse anti-GST antibodies (Transgene, HT601, 1:1000) were added for 45 min at 37 °C. Followed by the addition of a HRP-conjugated goat anti-mouse secondary antibody (Jackson ImmunoResearch Laboratories, 115–035-174, 1:20000) for 30 min at 37 °C. The plates were washed 3 times and incubated for 30 min with TMB. Optical density at 450 nm was measured in a microtiter plate reader.

### Inhibition of rabbit RBC hemolysis

Erythrocytes were collected and processed as described above. Antibodies (YG8–1, YG8–2 and YG8–3) were serially diluted and mixed with recombinant HlgAB (0.6 nM) or HlgBC (1.2 nM). After incubation at RT for 30 min, the mixture was incubated with 2% (*v*/v) solution of erythrocytes at 37 °C for 1 h. The optical density at 405 nm of cell supernantant was determined by a microplate reader. Percent inhibition of toxin activity was calculated using the following formula: percent inhibition = [(normal activity-inhibited activity)/(normal activity)] × 100. Data were analyzed by nonlinear regression analysis using Prism 6 (GraphPad).

### Murine model challenged with HlgAB

HlgABwas incubated with serially diluted YG8–2 for 30 min. Female 6–8 weeks old BALB/c mice (6 mice/group) were anesthetized and then retroorbitally challenged with toxin (1 μg/mouse) or the mixture of toxin and antibodies. Survival rate was assessed at different time points following bacterial challenge. Statistical analysis was performed by analysis of survival curves by the Logrank (Mantel-Cox) test using Prism 6 (GraphPad).

### Murine peritonitis model

Female 6–8 weeks old BALB/c mice (14 mice/group) were injected intraperitoneally with YG8–2 (10 μg/mouse or 100 μg/mouse) at 4 h before the bacterial challenge, then animals were challenged intraperitoneally with 0.5 ml *S. aureus* suspension (1.5 × 10^8^ CFU/ml). At 12 h post-challenge, the number of bacteria in peripheral blood was evaluated. And survival rate was assessed at different time points for 60 h after bacterial challenge. Then mice were sacrificed and the kidneys were harvested and homogenized. Homogenates were serially diluted and plated on BHI plates for CFU enumeration. Statistical analysis was performed by analysis of survival curves by the Logrank (Mantel-Cox) test using Prism 6 (GraphPad).

### Statistical analysis

All experiments were repeated at least three times and the data were presented as the mean ± SD unless noted otherwise. All quantitative were evaluated using a two-tailed Student’s t test. A *P*-value of less than 0.05 was considered to be statistically significant. GraphPad Prism software was used for statistical analyses.

## Additional files

Additional file 1: **Figure S1.** HlgABC, LukSF, and LukED proteins were expressed and analyzed by SDS-PAGE and Coomassie blue staining. (PDF 231 kb)

Additional file 2: **Figure S2**. Selection of HlgB-neutralizing antibodies from a naïve human Fab phage library. After three rounds of panning, 168 phage clones were further determined by ELISA, and 16 clones were then subjected to nucleotide sequencing (group 1: OD_450nm_ ≤ 0.4; group 2: 0.4 < OD4_50nm_ < 0.8; group 3: OD_450nm_ ≥ 0.8). (PDF 16 kb)

Additional file 3: **Figure S3.** YG8–1 **(a)**, YG8–2 **(b)**, and YG8–3 **(c)** were expressed and analyzed by reducing and nonreducing SDS-PAGE. (PDF 288 kb)

Additional file 4: **Figure S4.** Antibody affinity of YG8–1, YG8–2, and YG8–3 to HlgB **(a)**, LukF **(b)**, and LukD **(c)** determined with surface plasmon resonance (BIAcore). Anti-human IgG antibodies were immobilized on the carboxymethylated dextran surface of a CM5 chip, and YG8–1, YG8–2, and YG8–3 were captured by the immobilized antibody. HlgB, LukF, and LukD were injected at the indicated concentrations. The data were analyzed using Biacore X100 Evaluation software. (PDF 592 kb)

Additional file 5: **Figure S5.** Sequence alignment of HlgB and other F-components showing the highest homology. Identical amino acids are indicated in black. (PDF 52 kb)

## Abbreviations

IC50: Half maximal inhibitory concentration
mAbs: Monoclonal antibodies
RBCs: Red blood cells
rRBCs: Rabbit red blood cells
*S. aureus*: *Staphylococcus aureus*
SPR: Surface plasmon resonance

## References

[1] DeLeo FR, Chambers HF (2009). Reemergence of antibiotic-resistant Staphylococcus aureus in the genomics era. J Clin Invest.

[2] Deleo FR, Otto M, Kreiswirth BN, Chambers HF (2010). Community-associated meticillin-resistant Staphylococcus aureus. Lancet.

[3] Oganesyan V, Peng L, Damschroder MM, Cheng L, Sadowska A, Tkaczyk C (2014). Mechanisms of neutralization of a human anti-α-toxin antibody. J Biol Chem.

[4] Thammavongsa V, Rauch S, Kim HK (2015). Missiakas DM& Schneewind O. protein A-neutralizing monoclonal antibody protects neonatal mice against Staphylococcus aureus. Vaccine.

[5] Prevost G, Cribier B, Couppie P, Petiau P, Supersac G, Finck-Barbancon V (1995). Panton-valentine leucocidin and gamma-hemolysin from Staphylococcus aureus ATCC 49775 are encoded by distinct genetic loci and have different biological activities. Infect. Immun.

[6] Smith M, Price S (1938). Staphylococcus γ-hemolysin. J Pathol Bacteriol.

[7] Vandenesch F, Lina G, Henry T (2012). Staphylococcus aureus hemolysins, bi-component leukocidins, and cytolytic peptides: a redundant arsenal of membrane-damaging virulence factors?. Front Cell Infect Microbiol.

[8] Alonzo F, Torres VJ (2014). The bicomponent pore-forming leucocidins of Staphylococcus aureus. Microbiol Mol Biol Rev.

[9] DuMont AL, Torres VJ (2014). Cell targeting by the Staphylococcus aureus pore-forming toxins: it’s not just about lipids. Trends Microbiol.

[10] DeLeo FR, Diep B, Otto M (2009). Host defense and pathogenesis in Staphylococcus aureus infections. Infect Dis Clin N Am.

[11] Malachowa N, Whitney AR, Kobayashi SD, Sturdevant DE, Kennedy AD, Braughton KR (2011). Global changes in Staphylococcus aureus gene expression in human blood. PLoS One.

[12] Nilsson IM, Hartford O, Foster T, Tarkowski A (1999). Alpha-toxin and gamma-toxin jointly promote Staphylococcus aureus virulence in murine septic arthritis. Infect Immun.

[13] Siqueira JA, Speeg-Schatz C, Freitas FI, Sahel J, Monteil H, Prevost G (1997). Channel-forming leucotoxins from Staphylococcus aureus cause severe inflammatory reactions in a rabbit eye model. J Med Microbiol.

[14] Supersac G, Piemont Y, Kubina M, Prevost G, Foster TJ (1998). Assessment of the role of gamma-toxin in experimental endophthalmitis using a hlg-deficient mutant of Staphylococcus aureus. Microb Pathog.

[15] Seilie ES, Bubeck Wardenburg J (2017). Staphylococcus aureus pore-forming toxins: the interface of pathogen and host complexity. Semin Cell Dev Biol.

[16] Spaan AN, Schiepers A, de Haas CJ, van Hooijdonk DD, Badiou C, Contamin H (2015). Differential interaction of the Staphylococcus toxins Panton-valentine Leukocidin and γ-Hemolysin CB with human C5a receptors. J Immunol.

[17] Kaneko J, Ozawa T, Tomita T, Kamio Y (1997). Sequential binding of Staphylococcus gamma-hemolysin to human erythrocytes and complex formation of the hemolysin on the cell surface. Biosci Biotechnol Biochem.

[18] Prevost G, Couppie P, Prevost P, Gayet S, Petiau P, Cribier B (1995). Epidemiological data on Staphylococcus aureus strains producing synergohymenotropic toxins. J Med Microbiol.

[19] Von Eiff C, Friedrich AW, Peters G, Becker K (2004). Prevalence of genes encoding for members of the staphylococcal leukotoxin family among clinical isolates of Staphylococcus aureus. Diagn Microbiol Infect Dis.

[20] Spaan AN, Vrieling M, Wallet P, Badiou C, Reyes-Robles T, Ohneck EA (2014). The staphylococcal toxins γ-Hemolysin AB and CB differentially target phagocytes by employing specific chemokine receptors. Nat Commun.

[21] Colin DA, Mazurier I, Sire S, Finck-Barban V (1994). Interaction of the two components of leukocidin from Staphylococcus aureus with human polymorphonuclear leukocyte membranes: sequential binding and subsequent activation. Infect Immun.

[22] Rauch S, DeDent AC, Kim HK, Bubeck Wardenburg J, Missiakas DM, Schneewind O (2012). Abscess formation and alpha-hemolysin induced toxicity in a mouse model of Staphylococcus aureus peritoneal infection. Infect Immun.

[23] Rasko DA, Sperandio V (2010). Anti-virulence strategies to combat bacteria-mediated disease. Nat Rev Drug Discov.

[24] Rouha H, Badarau A, Visram ZC, Battles MB, Prinz B, Magyarics Z (2015). Five birds, one stone: neutralization of α-hemolysin and 4 bi-component leukocidins of Staphylococcus aureus with a single human monoclonal antibody. MAbs.

[25] Maynard JA, Maassen CB, Leppla SH, Brasky K, Patterson JL, Iverson BL (2002). Protection against anthrax toxin by recombinant antibody fragments correlates with antigen affinity. Nat Biotechnol.

[26] DuMont A. L., Yoong P., Day C. J., Alonzo F., McDonald W. H., Jennings M. P., Torres V. J. (2013). Staphylococcus aureus LukAB cytotoxin kills human neutrophils by targeting the CD11b subunit of the integrin Mac-1. Proceedings of the National Academy of Sciences.

[27] Spaan AN, Henry T, van Rooijen WJM, Perret M, Badiou C, Aerts PC (2013). The staphylococcal toxin panton-valentine leukocidin targets human c5a receptors. Cell Host Microbe.

[28] Reyes-Robles Tamara, Alonzo Francis, Kozhaya Lina, Lacy D. Borden, Unutmaz Derya, Torres Victor J. (2013). Staphylococcus aureus Leukotoxin ED Targets the Chemokine Receptors CXCR1 and CXCR2 to Kill Leukocytes and Promote Infection. Cell Host & Microbe.

[29] Alonzo F, Kozhaya L, Rawlings SA, Reyes-Robles T, DuMont AL, Myszka DG (2013). CCR5 is a receptor for Staphylococcus aureus leukotoxin ED. Nature.

[30] Hua L, Hilliard JJ, Shi Y, Tkaczyk C, Cheng LI, Yu X (2014). Assessment of an anti-alpha-toxin monoclonal antibody for prevention and treatment of Staphylococcus aureus-induced pneumonia. Antimicrob Agents Chemother.

[31] Foletti D, Strop P, Shaughnessy L, Hasa-Moreno A, Casas MG, Russell M (2013). Mechanism of action and in vivo efficacy of a human-derived antibody against Staphylococcus aureus alpha-hemolysin. J Mol Biol.

[32] Thomsen IP, Sapparapu G, James DBA, Cassat JE, Nagarsheth M, Kose N (2017). Monoclonal antibodies against the Staphylococcus aureus Bicompent Leukotoxin AB isolated following invasive human infection reveal diverse binding and modes of action. J Infect Dis.

[33] Badarau A, Rouha H, Malafa S, Battles MB, Walker L, Nielson N (2016). Context matters: the importance of dimerization-induced conformation of the LukGH leukocidin of Staphylococcus aureus for the generation of neutralizing antibodies. MAbs.

[34] Rouha H, Weber S, Janesch P, Maierhofer B, Gross K, Dolezilkova I (2018). Disarming Staphylococcus aureus from destroying human cells by simultaneously neutralizing six cytotoxins with two human monoclonal antibodies. Virulence.

[35] Diep BA, Le VT, Visram ZC, Rouha H, Stulik L, Dip EC (2016). Improved protection in a rabbit model of community-associated methicillin-resistant Staphylococcus aureus necrotizing pneumonia upon neutralization of Leukocidins in addition to alpha-Hemolysin. Antimicrob Agents Chemother.

[36] Yan J, Han D, Liu C, Gao Y, Li D, Liu Y (2017). Staphylococcus aureus VraX specifically inhibits the classical pathway of complement by binding to C1q. Mol Immunol.

[37] Cooper LZ, Madoff MA, Weinstein L (1996). Heat stability and species range of purified staphylococcal alpha-toxin. J Bacteriol.

[38] Kontermann R, Dübel S. Antibody engineering. 2010; Vol. 2. Springer.

