# Supplementary figures and images for: Novel human monoclonal antibodies targeting the F subunit of leukocidins reduce disease progression and mortality caused by Staphylococcus aureus
Source: BMC Microbiol. 2018 Nov 12;18:181. doi: 10.1186/s12866-018-1312-7 (PMC6233355; doi:10.1186/s12866-018-1312-7)

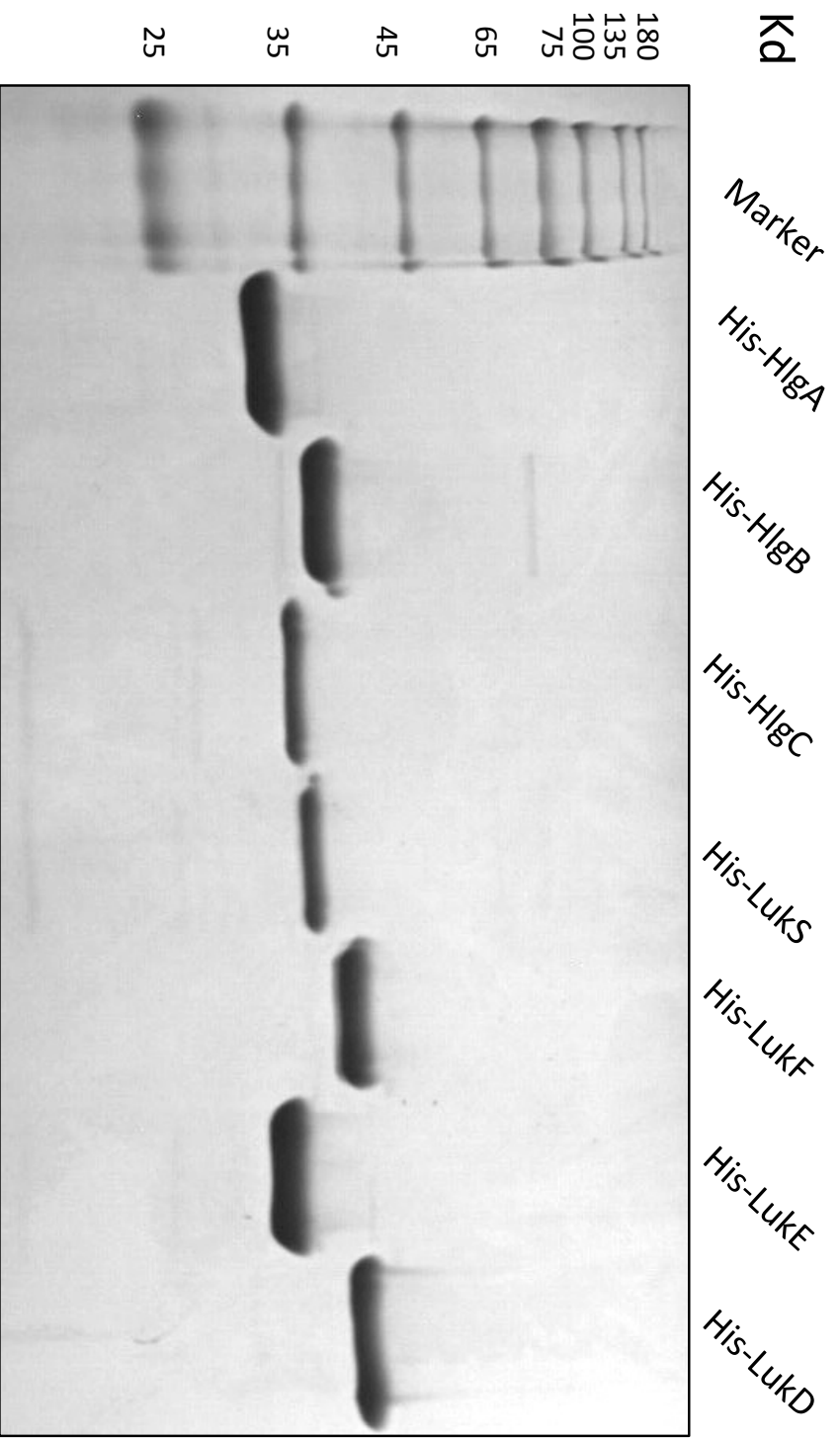

Supplement: Supplementary file 1 — Figure S1. HlgABC, LukSF, and LukED proteins were expressed and analyzed by SDS-PAGE and Coomassie blue staining. (PDF 231 kb) [file 12866_2018_1312_MOESM1_ESM.pdf]

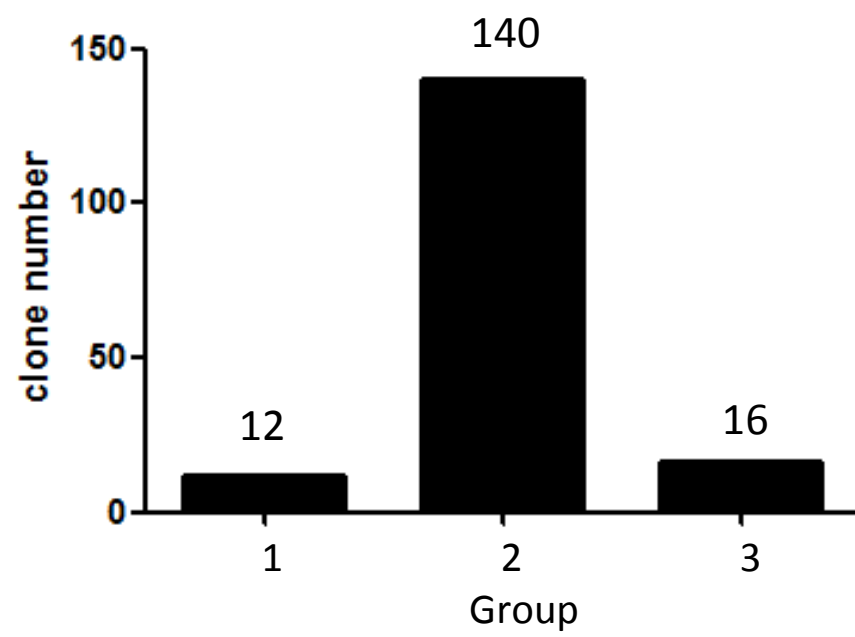

Supplement: Supplementary file 2 — Figure S2. Selection of HlgB-neutralizing antibodies from a naïve human Fab phage library. After three rounds of panning, 168 phage clones were further determined by ELISA, and 16 clones were then subjected to nucleotide sequencing (group 1: OD450nm ≤ 0.4; group 2: 0.4 < OD450nm < 0.8; group 3: OD450nm ≥ 0.8). (PDF 16 kb) [file 12866_2018_1312_MOESM2_ESM.pdf]

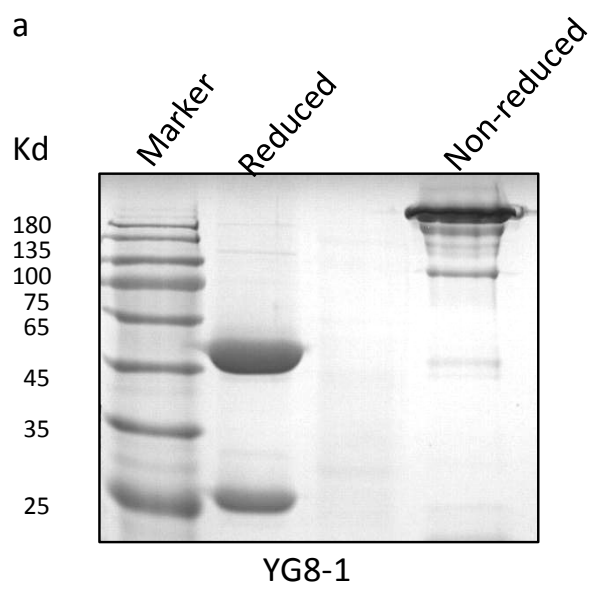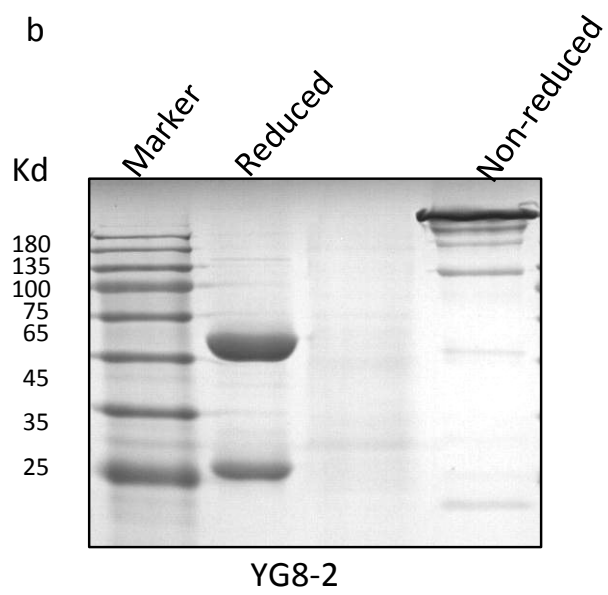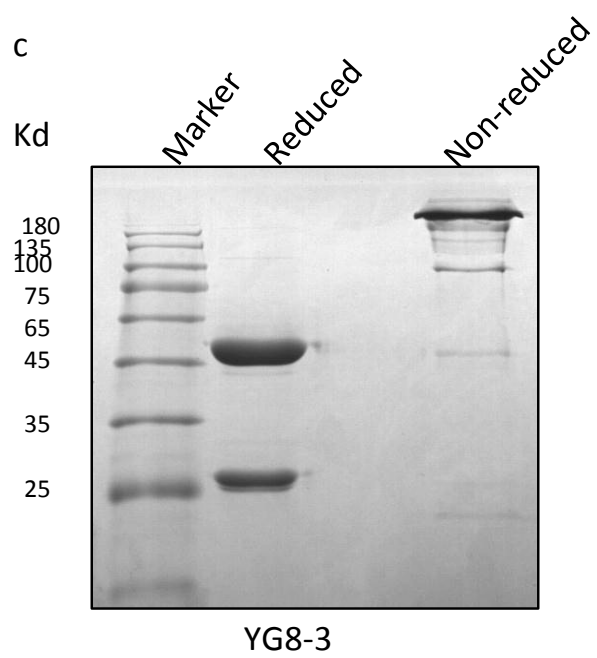

Supplement: Supplementary file 3 — Figure S3. YG8–1 (a), YG8–2 (b), and YG8–3 (c) were expressed and analyzed by reducing and nonreducing SDS-PAGE. (PDF 288 kb) [file 12866_2018_1312_MOESM3_ESM.pdf]

a

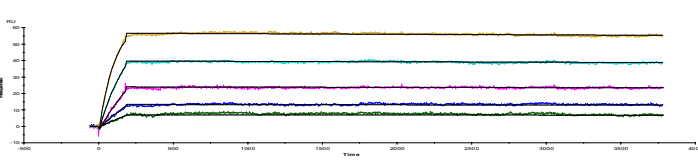

YG8-1

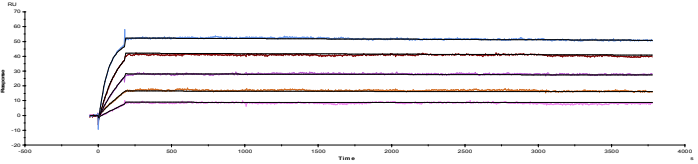

YG8-2

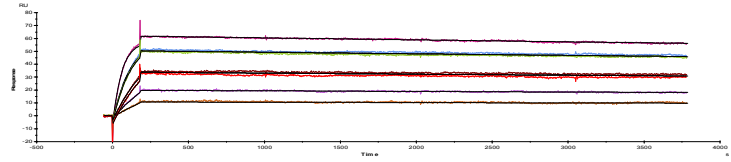

YG8-3

b

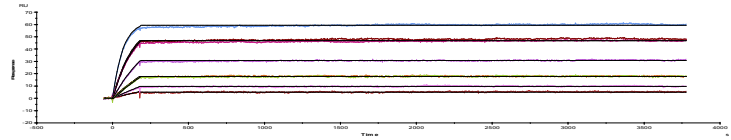

YG8-1

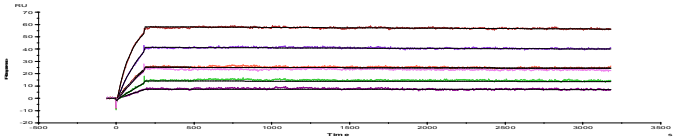

YG8-2

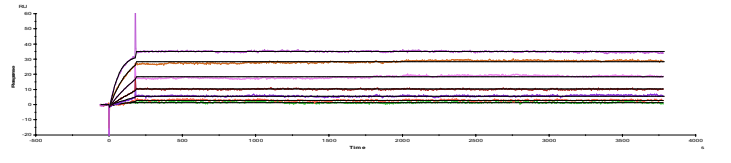

YG8-3

c

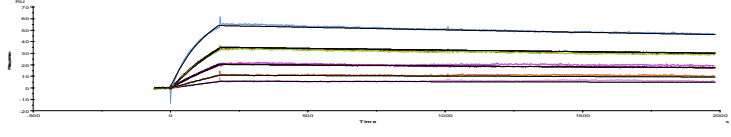

YG8-1

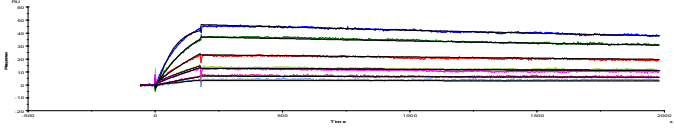

YG8-2

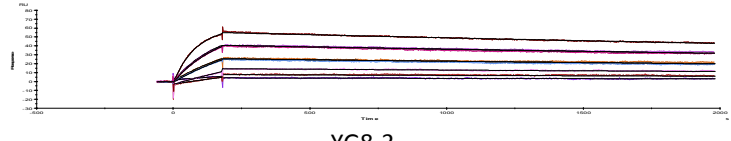

YG8-3

Supplement: Supplementary file 4 — Figure S4. Antibody affinity of YG8–1, YG8–2, and YG8–3 to HlgB (a), LukF (b), and LukD (c) determined with surface plasmon resonance (BIAcore). Anti-human IgG antibodies were immobilized on the carboxymethylated dextran surface of a CM5 chip, and YG8–1, YG8–2, and YG8–3 were captured by the immobilized antibody. HlgB, LukF, and LukD were injected at the indicated concentrations. The data were analyzed using Biacore X100 Evaluation software. (PDF 592 kb) [file 12866_2018_1312_MOESM4_ESM.pdf]
