# Supplementary material for: Novel human monoclonal antibodies targeting the F subunit of leukocidins reduce disease progression and mortality caused by Staphylococcus aureus
Source: BMC Microbiol. 2018 Nov 12;18:181. doi: 10.1186/s12866-018-1312-7 (PMC6233355; doi:10.1186/s12866-018-1312-7)
Supplement: Supplementary file 5 — Figure S5. Sequence alignment of HlgB and other F-components showing the highest homology. Identical amino acids are indicated in black. (PDF 52 kb) [file 12866_2018_1312_MOESM5_ESM.pdf]

```
hlgb      MKMNKLVKSSVATSMALLLLSGTANAEGKITPVSVKKVDD     40
lukd      MKIEKLGKSSVASSIALLLLSNTVDAAQNITPKREKKVDD     40
lukf      MKMNKLVKSSVATSMALLLLSGTANAEGKITPVSVKKVDD     40
Consensmk  kl kssva s allll s t  a   itp   kkvdd

hlgb      KVTLYKTTATADSDKFKISQILTFNFIKDKSYDKDTLVLK     80
lukd      KITLYKTTATSDNDKLNIFQILTFNFIKDKSYDKDTLVLK     80
lukf      KVTLYKTTATADSDKFKISQILTFNFIKDKSYDKDTLVLK     80
Consenskstlykttat d dk  i qiltfnfikdksydkdtlvlk

hlgb      ATGNINSGFVKPNPNDYDFSKLYWGAKYNVSISSQSNDSV     120
lukd      AAGNINSGYKNSNPKDYNYSQFYWGGKYNVSVSSESNDAV     120
lukf      ATGNINSGFVKPNPNDYDFSKLYWGAKYNVSISSQSNDSV     120
Consensasgninsg     np dy  s   ywg kynvs ss snd v

hlgb      NVVDYAPKNQNEEFQVQNTLGYTFGGDISISNGLSGGLNG     160
lukd      NVVDYAPKNQNEEFQVQQTLGYSYGGDINISNGLSGGLNG     160
lukf      NAVDYAPKNQNEEFQVQNTLGYTFGGDISISNGLSGGLNG     160
Consensnsvdyapknqneefqvq tlgy  ggdi isnglsgglng

hlgb      NTAFSETINYKQESYRTTLSRNTNYKNVGWGVEAHKIMNN     200
lukd      SKSFSETINYKQESYRTTIDRKTNHKSIGWGVEAHKIMNN     200
lukf      NTAFSETINYKQESYR.TLSRNTNYKNVGWGVEAHKIMN.     198
Consensus fsetinykqesyr t  r tn k  gwgveahkimn

hlgb      GWGPYGRDSFHPTYGNELFLAGRQSSAYAGQNFIAQHQMP     240
lukd      GWGPYGRDSYDPTYGNELFLGGDKSSSNAGQNFLPTHQIP     240
lukf      GWGPYGRDSFHPTYGNELFLAGRQSSAYAGQNFIAQHQMP     238
Consensgwgpygrds  ptygnelfl g  ss  agqnf   hq p

hlgb      LISRSNFNPEFLSVLSHRQDGAKKSKITVTYQREMDLYQI     280
lukd      LIARGNFNPEFISVLSHKLFDTKKSKIKVTYQREMDRYTN     280
lukf      LISRSNFNPEFLSVLSHRQDRAKKSKITVTYQREMDLYQI     278
Consensll  r nfnpef svlsh     kkski vtyqremd y

hlgb      RWNGFYWAGANYKNFKTRTFKSTYEIDWENHKVKILDTKE     320
lukd      QWNRSHWVGNNYKNQNTVTFTSTYEVDWQNILLKLIGTDS     320
lukf      RWNGFYWAGANYKNFKTRTFKSTYEIDWENHKVKILDTKE     318
Consensuwn   w g nykn  t tf stye dw n   kl  t

hlgb      TENNK.                                       325
lukd      KETNPG                                       326
lukf      TENNK.                                       323
Consensue n
```
